# Inactivation of the Medial-Prefrontal Cortex Impairs Interval Timing Precision, but Not Timing Accuracy or Scalar Timing in a Peak-Interval Procedure in Rats

**DOI:** 10.3389/fnint.2018.00020

**Published:** 2018-06-25

**Authors:** Catalin V. Buhusi, Marcelo B. Reyes, Cody-Aaron Gathers, Sorinel A. Oprisan, Mona Buhusi

**Affiliations:** ^1^Interdisciplinary Program in Neuroscience, Department of Psychology, USTAR BioInnovations Center, Utah State University, Logan, UT, United States; ^2^Department of Neurosciences, Medical University of South Carolina, Charleston, SC, United States; ^3^Department of Physics and Astronomy, College of Charleston, Charleston, SC, United States

**Keywords:** interval timing, medial prefrontal cortex (mPFC), pharmacology, muscimol, computational modeling, rats, Sprague-Dawley, Striatal Beat Frequency model

## Abstract

Motor sequence learning, planning and execution of goal-directed behaviors, and decision making rely on accurate time estimation and production of durations in the seconds-to-minutes range. The pathways involved in planning and execution of goal-directed behaviors include cortico-striato-thalamo-cortical circuitry modulated by dopaminergic inputs. A critical feature of interval timing is its scalar property, by which the precision of timing is proportional to the timed duration. We examined the role of medial prefrontal cortex (mPFC) in timing by evaluating the effect of its reversible inactivation on timing accuracy, timing precision and scalar timing. Rats were trained to time two durations in a peak-interval (PI) procedure. Reversible mPFC inactivation using GABA agonist muscimol resulted in decreased timing precision, with no effect on timing accuracy and scalar timing. These results are partly at odds with studies suggesting that ramping prefrontal activity is crucial to timing but closely match simulations with the Striatal Beat Frequency (SBF) model proposing that timing is coded by the coincidental activation of striatal neurons by cortical inputs. Computer simulations indicate that in SBF, gradual inactivation of cortical inputs results in a gradual decrease in timing precision with preservation of timing accuracy and scalar timing. Further studies are needed to differentiate between timing models based on coincidence detection and timing models based on ramping mPFC activity, and clarify whether mPFC is specifically involved in timing, or more generally involved in attention, working memory, or response selection/inhibition.

## Introduction

Animal and human studies have revealed critical roles of the *medial prefrontal cortex* (mPFC; and its human counterpart, dorso-lateral prefrontal cortex) in a variety of cognitive processes from attention (Arnsten, 2009; Paneri and Gregoriou, 2017), working memory (Funahashi, 2017; Murray et al., 2017; Spaak et al., 2017), inhibitory control (Jonkman et al., 2009) or habit formation (Yin and Knowlton, 2006; Limpens et al., 2015) to more complex functions such as planning and decision making (Dixon and Christoff, 2014; Padoa-Schioppa and Conen, 2017) and action selection (Matsumoto et al., 2003; Ridderinkhof et al., 2004) or cognitive flexibility (Robbins, 2007; Kehagia et al., 2010). These functions are based on the extensive interconnectivity of the mPFC with other cortical (Crowe et al., 2013; Phillips et al., 2014) and subcortical regions, such as the thalamus, amygdala, hippocampus and striatum (Kesner and Churchwell, 2011).

Time perception and processing are essential for planning and decision making (Buhusi and Meck, 2005; Meck et al., 2012; Bermudez and Schultz, 2014; Finnerty et al., 2015; Kirkpatrick and Balsam, 2016), thus many studies have focused on evaluating how interval timing is perceived, encoded and processed in the brain. Initial lesion studies have questioned a role of the mPFC in memory for duration (Jackson et al., 1998) or revealed an initial impairment of acquisition of a timed response, that could be overcome through extensive training (Dietrich and Allen, 1998). More recent studies, using pharmacological inactivation of the mPFC (Kim et al., 2009) or electrophysiological recordings of mPFC neurons (Kim et al., 2013) in a temporal bisection procedure suggest that mPFC is involved in the discrimination of time durations. Also, changes in mPFC neurons firing patterns have been identified during the performance of timed behavior (Niki and Watanabe, 1979; Xu et al., 2014). However, the procedures used in these studies are relatively different from standard timing tasks and are apt to introduce artifacts. For example, using fixed-interval trials in Emmons et al. (2017) is likely to introduce artifacts related to expectation of reward; moreover, fixed-interval trials cannot be used to evaluate the subjective criterion the rats have acquired, this can be done only in *peak-interval* (PI) trials (see below). Similarly, introducing extraneous response requirements (e.g., remaining in the port for the duration followed by exit in Xu et al., 2014) is likely to introduce artifacts related to response inhibition followed by action (for a similar argument see also, Namboodiri and Hussain Shuler, 2014).

The standard *PI* procedure has several distinct advantages over the procedures used in the above studies. First, by examining timing in “peak”, non-reinforced, probe trials, one can isolate interval timing without interference from other processes, e.g., reward. Moreover, in the PI procedure one can simultaneously estimate both timing accuracy (peak time) and precision (width of the response function; Buhusi and Meck, 2010). Finally, when introducing distractors, one can also estimate memory for time and/or attention to time (Buhusi and Meck, 2000, 2009; Buhusi, 2003). Here, we examined the role of the mPFC in timing behavior by evaluating the effect of its reversible inactivation on timing accuracy, timing precision and scalar timing, in a PI procedure.

## Materials and Methods

### Subjects

Twelve naïve Sprague-Dawley male rats, 3 months old at the beginning of the experiment, were housed individually in a temperature-controlled room, under a 12/12 h light-dark cycle, with water given *ad libitum*. Rats were maintained at 85% of their *ad libitum* weight by restricting access to food (Rodent Diet 5001, PMI Nutrition International Inc., Brentwood, MO, USA). All experimental procedures were conducted in accordance with the National Institutes of Health’s Guide for the Care and Use of Laboratory Animals (1996) and approved by the Institutional Animal Care and Use Committee of the Medical University of South Carolina.

### Apparatus

The apparatus consisted of 12 standard rat operant chambers (MED Associates, St. Albans, VT, USA) housed in sound attenuating cubicles. Chambers were equipped with two fixed levers situated on the front wall of the chamber. According to the schedule, 45 mg precision food pellets (BioServ, Frenchtown, NJ, USA) were delivered in a food cup situated on the front wall, 1 cm above the grid floor, between the two levers, by a pellet dispenser. The to-be-timed visual stimuli were two cue lights located about 7 cm above the levers. A 66-dB background sound produced by a ventilation fan was present throughout the session.

### Dual Peak-Interval (PI) Training

Rats were shaped to lever press, after which they were randomly assigned to two groups trained in a dual PI procedure with different timing criteria: group G1–10/20 (*n* = 6) was trained in a dual PI procedure with criteria 10 s and 20 s, while group G2–20/40 (*n* = 6) was trained in a dual PI procedure with criteria 20 s and 40 s, as in Reyes and Buhusi (2014). On average, each session contained 50% fixed-interval trials, in which rats were presented with one of the cue lights and reinforced for the first lever press (on the associated lever) after the specific criterion, and 50% PI trials, in which rats were presented with one of the cue lights for three times the criterion time but were not reinforced for lever pressing. The levers and cue lights associated with the short and long criteria were counterbalanced among rats. Both levers were permanently available for pressing, but in each trial only one cue light and one lever were active. Trials were presented in a pseudo-random manner, separated by random inter-trial intervals (ITIs), ranging from 2.5 to 3.5 times the criterion associated with the current trial, uniformly distributed.

### Surgery

Surgery and drug infusion procedures closely followed those in Matthews et al. (2012): briefly, during aseptic surgery under isoflurane anesthesia, 26-gauge bilateral cannula guides (PlasticsOne, Roanoke, VA, USA) were implanted aiming at the medial prefrontal cortex (AP 3.2 mm, ML ± 0.6 mm, DV −3.5 mm; Paxinos and Watson, 1998) and embedded in dental cement. Rats were given a week to recover from surgery before retraining. Rats were re-trained before any local infusions began.

### Local Medial-Prefrontal Cortex (mPFC) Infusions

Cannulae injectors aiming at mPFC were lowered into the cannula guides, extending 1 mm below the guides. Rats received intracranial injections of GABA-A receptor agonist muscimol in a saline solution. Rats received microinjections of 0.5 μL of 0 mM, 0.11 mM, or 1 mM muscimol solution, equivalent to 0, 7 ng, or 63 ng muscimol, respectively. Infusions were done bilaterally, at a rate of 0.25 μL/min over 2 min, followed by a 2 min interval to allow the drug to infuse the tissue; 15 min afterwards, rats were placed into the chambers for testing in the dual PI paradigm. Infusion sessions were separated by at least two no-drug dual-PI sessions. The order of drug doses was counterbalanced between animals.

### Histology

**Figure 1:**
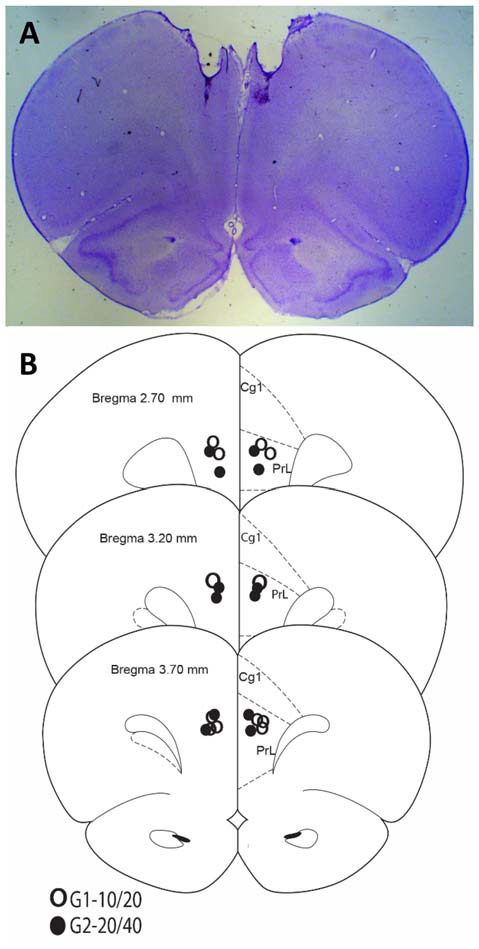
Histological analyses. **(A)** Representative Nissl-stained section. **(B)** Cannula placements in the present experiment.

Rats were anesthetized with isoflurane overdose and transcardially perfused with formalin. Brains were collected and sectioned on a vibratome. Sixty-micron sections were placed on slides and stained with cresyl violet for histological analyses. Figure 1 indicates that all rats in both groups were infused in the target area (mPFC).

### Data Collection and Analysis

Data collection and analysis closely follows (Matthews et al., 2012; Reyes and Buhusi, 2014). Briefly, lever presses were recorded in real time by a MED Associates system running a MED-PC software system (MED Associates, 1999). Lever presses from dual PI trials with drug infusion were used to estimate timing accuracy (peak time) for each rat as in Buhusi and Meck (2000) and peak response rate. Response functions were then normalized in time and in amplitude as in Buhusi et al. (2009). To quantify scalar property, we estimated the degree of superposition of the normalized response curves using the *η*^2^ index as in Brown et al. (2007) and Buhusi et al. (2009). An *η*^2^ equal to 1 indicates perfect superposition, *η*^2^ around or larger than 0.9 indicates a very good degree of superposition (scalar timing), while an *η*^2^ around or lower than 0.7 indicates rather poor superposition. To further investigate the effect the drug on the dynamics of timing behavior, individual-trial analyses were performed as described in Church et al. (1994) and Swearingen and Buhusi (2010). Analysis algorithms described in Swearingen and Buhusi (2010) were used to extract the start and stop times during individual trials. The dependent variables peak time, width of function, start time, stop time, response rate and *η*^2^ were submitted to mixed analysis of variances (ANOVAs) with independent between-subject variable group (G1–10/20, G2–20/40) and within-subject variables trial type (short, long) and drug dose (0, 7, 63 ng/side muscimol). Statistical tests were evaluated at a significance level of 0.05.

## Results

### Baseline Timing

**Figure 2:**
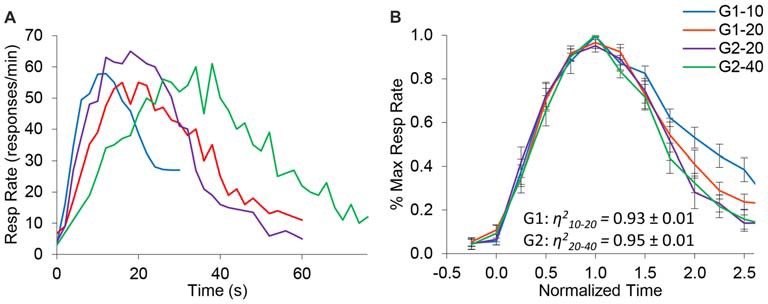
Response functions in the dual peak-interval (PI) procedure under saline condition. **(A)** Average response rates (resp/min) in the saline condition by group and criterion. **(B)** Average response rates (±SEM) normalized in amplitude and time. The degree of superposition between normalized functions is indicated by the superposition index *η*^2^, which shows a very high compliance with scalar timing.

Figure 2A shows the average response rates in the dual PI (non-reinforced) trials during the baseline (control) saline condition. Rats clearly produced distinct peaks for each of the trial types, showing that their timing was accurate. Analyses of peak time indicated a significant effect for group (*F*_(1,10)_ = 49) and trial type (*F*_(1,10)_ = 72.1), and a significant group × trial interaction (*F*_(1,10)_ = 7.4). Response functions were normalized in amplitude and in time, as shown in Figure 2B. To quantify scalar property, we estimated the degree of superposition of the normalized response curves for each rat between the short and long trials using the *η*^2^ superposition index as in Brown et al. (2007) and Buhusi et al. (2009). No significant differences in *η*^2^ were found between groups (*F*_(1,10)_ = 0.39). Estimated *η*^2^ was 0.93 ± 0.01 in group G1, and 0.95 ± 0.01 in group G2, indicating a very high degree of superposition (scalar timing). Results indicate very good superposition of response function in both groups and on both durations, thus indicating that rats acquired the timing task and their timing was accurate and scalar in the baseline (saline) condition.

### General Effect of mPFC Inactivation

**Figure 3:**
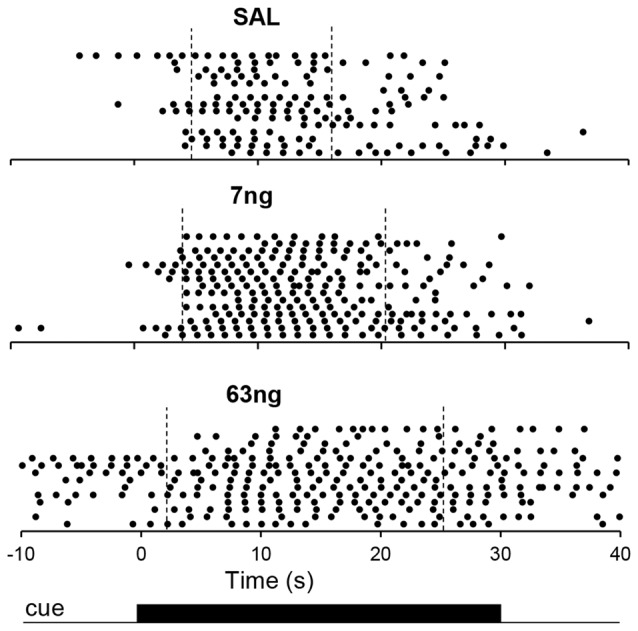
Timing under muscimol mPFC inactivation in a representative rat. Panels show raster plots of responding in during 10-s PI trials by a representative rat before, during and after the timing cue under saline (upper panel), 7 ng muscimol (middle panel) and 63 ng muscimol (lower panel). The presentation of the timing cue (10-s peak trial) is indicated at the bottom of the figure (see text for details).

Figure 3 shows response raster plots of a representative rat in a 10-s PI session under the three drug conditions, before, during and after the timing cue. Vertical dotted lines indicate the estimated start times (left) and stop times (right). The upper panel indicates that rat’s responses were tightly grouped around the criterion duration (10 s), although one can observe trial-to-trial variations. The middle panel indicates that under the 7 ng muscimol dose, the rat’s rate of response increased: while responses remained grouped around the criterion interval, the rat responded earlier (start time decreased) and later (stop time increased). Finally, the lower panel indicates that under the 63 ng dose, rat’s response rate increased further not only during the timing cue but also before and after (during the ITI), although most responses were emitted still during the cue, before and after the criterion duration: the start time decreased further, but remained larger than 0 s, the start of the timing cue, and the stop time increased further but remained below 30 s, the end of the timing cue in a 10-s peak trial. These features were further explored and quantified as described below.

### Specific Effects of mPFC Inactivation on Interval Timing

**Figure 4:**
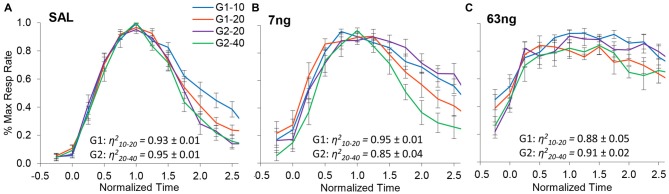
mPFC inactivation preserves accurate, scalar timing. Average normalized response functions (±SEM) in the dual PI procedure under muscimol mPFC inactivation by group and trial type: saline **(A)**, 7 ng/side muscimol **(B)**, and 63 ng/side muscimol **(C)**. The average superposition index *η*^2^_short-long_ (±SEM) is provided for each group and drug condition, indicating a very high degree of superposition (scalar timing).

Figure 4 shows normalized response rates under saline (left panel), 7 ng/side muscimol (center panel), and 63 ng/side (right panel) by group and trial type. A visual inspection of panels of Figure 4 indicates that despite mPFC inactivation timing continued to be accurate (response rate peaked about normalized time 1.0) and scalar (curves superimposed well in normalized time irrespective of drug dose). Figure 4 indicates that mPFC inactivation preserved accurate timing (had no significant effect on timing accuracy, *F*_(2,11)_ = 1.2), but impaired the precision of timing (significantly increased the width of the PI functions, *F*_(2,11)_ = 12.7). To evaluate the effect of mPFC inactivation on scalar property, we estimated the degree of superposition between short and long trials for each rat at each muscimol dose, using the *η*^2^ superposition index (Brown et al., 2007; Buhusi et al., 2009). Rats in all groups exhibited very large *η*^2^_short-long_: the average *η*^2^_short-long_ was 0.91 ± 0.01 under saline, 0.90 ± 0.02 under 7 ng muscimol, and 0.89 ± 0.03 under 63 ng muscimol, indicative of very good superposition at all muscimol doses. Analyses of variance failed to indicate significant effects of group, dose, or interactions on the degree of superposition (all *F*s < 2.65). In summary, in the conditions of this study mPFC inactivation preserved scalar timing irrespective of muscimol dose.

**Figure 5:**
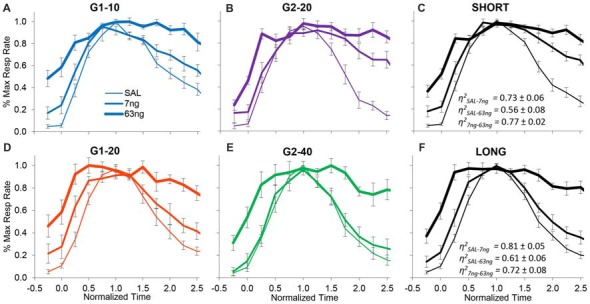
mPFC inactivation preserves accurate timing but decreases timing precision. Average normalized response functions (±SEM) during mPFC inactivation by muscimol dose****, by group (G1 Panels **A,D**, G2 Panels **B,E**), and averaged by trial type: short trials G1–10 and G2–20 (Panel **C**), and long trials G1–20 and G2–40 (Panel **F**). SAL = saline (0 ng/side muscimol). The average superposition index *η*^2^ (±SEM) indicates a relatively low degree of superposition between functions at different drug doses.

On the other hand, when the degree of superposition was estimated for each rat between drug doses, results were very much different. The normalized response curves were redrawn in Figure 5 by group (G1: panels **A,D**; G2: panels **B,E**), and averaged by trial type: short trials (G1–10 and G2–20, panel **C**), and long trials (G1–20 and G2–40, panel **F**). A visual inspection of Figure 5 indicates that mPFC inactivation did not affect accuracy (response peaked about normalized time 1.0), but impaired the precision of timing (significantly increased the width of the PI functions, *F*_(2,11)_ = 12.7).

To further evaluate this effect, for each subject we estimated the degree of superposition between response curves at different muscimol doses. Irrespective of group, rats exhibited relatively low superposition between response curves at different doses. Panels **C,F** of Figure 5 show the average superposition indices *η*^2^ between response curves at different doses for short trials (G1–10 and G2–20, panel **C**), and long trials (G1–20 and G2–40, panel **F**). The average *η*^2^_SAL-7 ng_ was 0.76 ± 0.05, the average *η*^2^_SAL-63 ng_ was 0.57 ± 0.06, and the average *η*^2^_7 ng-63 ng_ was 0.79 ± 0.04, indicative of poor superposition between muscimol doses. In summary, in all groups and conditions, gradual mPFC inactivation altered the response curves by making the curves wider but peaking about the criterion interval. To quantify the increase in the width of the response functions with the muscimol dose, and to evaluate whether the widening of the response curve was a phenomenon happening at the trial level or whether it was an artifact of averaging over trials, we further performed individual-trial analyses as described below.

**Figure 6:**
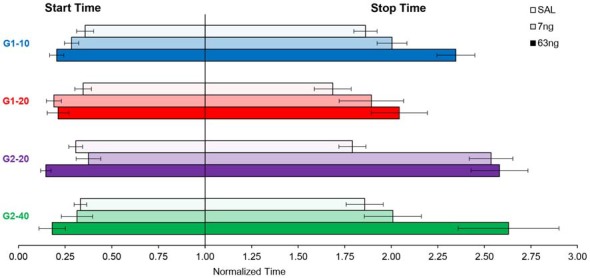
mPFC inactivation decreased start times and increased stop times in individual trials. Average (±SEM) estimated start times (left) and stop times (right) by group and criterion time.

In Figures 2, 4, 5 data were shown as average response curves, averaged over trials. On the other hand, in each trial, responses are thought to be better approximated by a low-high-low function (Church et al., 1994). Therefore, to gain further understanding of the effect of the mPFC inactivation on the dynamics of timing behavior, individual-trial analyses were performed as described in Swearingen and Buhusi (2010) to extract the start and stop times during individual trials. The *start time* is the time point at which there is a significant increase in response rate during the trial (at the transition from the low to high states). The *stop time* is the point during the trial at which there is a significant decrease in response rate (at the transition from the high to low states). The estimated start and stop times are shown in Figure 6, which also shows the “high response state” in between the start and stop times. A visual evaluation of the figure indicates that in all groups and conditions, the increase in muscimol dose resulted in start times decreasing and stop times increasing, compatible with the response curves becoming wider in Figure 5. Analyses of the estimated start times (Figure 6, left) and stop times (Figure 6, right) indicated a significant effect of the muscimol dose on both the start times (*F*_(2,20)_ = 5.38) and stop times (*F*_(2,20)_ = 12.2). The effect size (% of explained variance in start and stop times) of the muscimol dose was 21% on the start times, and 31% on the stop times, which are medium-to-large effects considering the design of the study involving 3 variables (group, trial type, dose) and their multiple interactions. Importantly, under all muscimol conditions the start times remained significantly larger than 0 (all one tail *t*s_(11)_ > 2.55), and start times remained significantly larger than 3 (all one tail *t*s_(11)_ > 1.37). These results suggest that mPFC inactivation resulted in a simultaneous decrease in start times and increase in stop times, such that the widening of the PI function shown in Figure 5 was not an artifact of averaging over trials.

### Specific Effects of mPFC Inactivation on Response Rates

**Figure 7:**
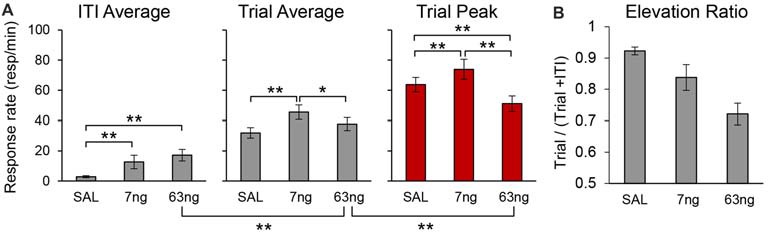
mPFC inactivation differentially alters response rates during the timed cue and the inter-trial interval (ITI) but does not impair cue-ITI discrimination. **(A)** Average peak response rates (peak), average response rates during the trial (trial), and average response rates during the ITI (±SEM) under saline (SAL), 7 ng/side muscimol, and 63 ng/side muscimol. **(B)** Average elevation ratio between the ITI and the trial (±SEM) under saline (SAL), 7 ng/side muscimol, and 63 ng/side muscimol. The elevation ratio was computed as the ratio between the average rate of response in the trial and the sum of the average rate during the trial and ITI. An elevation ratio of 0.5 would indicate that rats do not discriminate the timing cue from the ITI. *p < 0.05; **p < 0.01.

To evaluate the effect of mPFC inactivation on the rate of responding for each individual rat we estimated peak responding (during the trial), the average rate of response during the trial, and the average rate of response during the ITI, in each drug condition. Figure 7A details the average peak, average trial response rate and average ITI response rate. Analyses indicated a significant effects of response rate measure (Peak, Trial, ITI; *F*_(2,22)_ = 187.58), muscimol dose (*F*_(2,22)_ = 7.87), and a measure × dose interaction (*F*_(4,44)_ = 11.09). *Post hoc* analyses (Fisher LSD test) indicated that the response rate significantly increased with the muscimol dose during the ITI, but that effects were mixed during the trial: relative to saline, the trial average response rate significantly increased at the 7 ng, but not at the 63 ng dose.

Most importantly, analyses of response rates support our previous findings that irrespective of drug dose, responding during the trial was not flat throughout the trial (which would indicate loss of temporal control): irrespective of drug dose, the peak rate was significantly higher than average trial rate (*F*_(1,11)_ = 163.95). Also, the rats clearly discriminated between the timed cue and the ITI: the average trial rate was significantly higher than average ITI rate (*F*_(1,11)_ = 183.57). *Post hoc* analyses (Fisher LSD test) indicated that the trial response rates were significantly higher than ITI response rates at all doses. For simplicity, Figure 7A shows only the significant difference between the ITI rate and the trial rate at the 63 ng dose.

To further substantiate the finding that, irrespective of drug dose, rats discriminated the timing cue from the ITI, for each individual and for each condition we computed an elevation ratio between the ITI and the trial, equal to the average rate of response in the trial divided by the sum of the average rate during the trial and ITI. An elevation ratio of 0.5 would indicate that rats do not discriminate the timing cue from the ITI. The average elevation ratio is shown in Figure 7B, which indicates that mPFC inactivation significantly decreased the elevation ratio (*F*_(2,20)_ = 20.50), but that the ratio remained well above 0.5 (all *t*s_(11)_ > 6.44). The decrease in elevation ratio is due in part to the increase in response rate in the ITI possibly due to disinhibition (Figure 7A), and in part to the decrease in response rates during the trial, which in turn is partly due to the spreading of responses during the trial: responses were generated earlier (smaller start times, Figure 6; see also Figure 3 for early responding in a representative rat) and persisted later (larger stop times, Figure 6; see also Figure 3 for late responding in a representative rat). For simplicity Figure 7A shows the significant difference between the ITI rate and the trial rate at the 63 ng dose—indicating that rats discriminated the trial from the ITI—and the significant difference between the trial rate and the peak rate at the 63 ng dose—indicating that the response at the 63 ng rats continued to be peak shaped. Taken together, these findings strongly suggest that mPFC inactivation did not result in loss of temporal control and did not abolish timing cue-ITI discrimination.

## Discussion

The role of the mPFC in timing behavior was evaluated by its reversible inactivation with GABA agonist muscimol (MUSC). Rather than globally inactivating mPFC with large muscimol doses, here our doses were rather small, which allowed us to evaluate the effect of mPFC inactivation on timing accuracy, scalar timing, timing precision and response rate in a PI procedure. Inactivation of mPFC failed to affect timing accuracy (Figures 2, 4, 5), preserved scalar timing (Figures 2, 4), but decreased timing precision (widened the PI functions) by decreasing the start times and increasing stop times (Figure 6) in individual trials.

These results were not simply due to an increase in response rates. Indeed, the rate of response during the ITI increased with increasing muscimol dose (Figure 7A): this effect can also be seen by comparing the response rate before the trial starts, i.e., before time zero (cue presentation) in Figures 4, 5. However, mPFC inactivation had mixed effects on responding during the trial: relative to saline it increased responding at the 7 ng dose but had no effect at the 63 ng dose (Figure 7A). These results are compatible with a general decrease in behavioral inhibition rather than impulsivity. For example, previous studies found that mPFC inactivation reduced inhibitory control but does not affect impulsivity in the delay discounting paradigm (Feja and Koch, 2014). Indeed, in our study mPFC inactivation resulted in smaller start times, but also larger stop times, a result compatible with loss of inhibitory control, but seemingly incompatible with impulsivity.

Importantly, results cannot be simply due to animals failing to differentiate trials from the ITI, as the elevation ratio between the ITI to the trial was significantly larger than 0.5 (Figure 7B). This effect can be seen in Figures 4, 5, which indicate a clear difference in response rates between PI trial and the ITI. Moreover, rats clearly responded with a bell-shaped PI function during the to-be-timed cue (see Figures 2, 4, 5, 7A). In summary, although mPFC inactivation resulted in a general increase in response rate during the ITI, it is unlikely this increase had a major effect on the shape of the response function during peak trials.

Data from the present study can be used to differentiate various hypotheses on time/temporal representations in mPFC. One such hypothesis is that timing relies critically on ramping neuronal activity in mPFC. Indeed, changes in mPFC neurons firing patterns have been identified during the performance of timed behavior (Niki and Watanabe, 1979; Xu et al., 2014). However, the procedures used in these studies are relatively different from the timing task used in our study, and are prone to introducing artifacts (for a discussion see Namboodiri and Hussain Shuler, 2014). Moreover, ramping neuronal activity in the mPFC has been also reported in simple reaction time tasks (Narayanan and Laubach, 2009) or during the delay in delayed-response tasks in primates (Quintana et al., 1988; Compte et al., 2003; Tsujimoto and Sawaguchi, 2004), where it was interpreted as related to inhibition of the response or to mnemonic processing of stimulus attributes, respectively, rather than timing *per se*. Therefore, it is unlikely that ramping neuronal activity in the mPFC specifically codes for time.

Moreover, it seems that in the conditions of the present experiment, the ramping neuronal activity hypothesis would generate different predictions than our observed results. Under the hypothesis that timing is solely coded by ramping mPFC activity, it seems that mPFC inactivation should have resulted in alterations in timing accuracy (e.g., delayed timing), although it could be argued that, due to the relatively small muscimol doses, one could expect a general decrease in firing rates but without changing the timing peaks of activity given. Most importantly, a general (albeit small) decrease in firing rates should move the start and stop times in the same direction (should increase both the start and stop times). Instead, in our study start times and stop times moved in opposite directions (Figure 6), suggesting that the results are partly at odds with predictions based on the assumption that time is coded solely by ramping mPFC activity. Nevertheless, at this time ramping neuron models have not been clearly extended to cortico-striatal circuits or muscimol, and thus interpretations based on these models are rather speculative. Therefore, other putative time coding mechanisms are worth considering.

One such form of nonlinear coding is coincidence detection of distributed neuronal inputs (Miall, 1989; Matell and Meck, 2004; Buhusi and Meck, 2005; Buhusi and Oprisan, 2013). For example, a description of the neurobiological mechanisms involved in interval timing is currently provided by the Striatal Beat-Frequency (SBF) model, which ascribes a role for detecting event durations to medium spiny neurons within the dorsal striatum (Matell and Meck, 2004; Buhusi and Meck, 2005), which become entrained to fire in response to oscillating, coincident cortical inputs that become active at previously trained event durations. An interesting feature of this model is that scalar property emerges in the model due to neural noise (Buhusi and Oprisan, 2013; Oprisan and Buhusi, 2013a,b, 2014). In regard to the current experiment, reversible mPFC inactivation resulted in a decrease in timing precision, which could be interpreted as reflecting an increase in neural noise. Previous computational modeling indicated that in the SBF model a decrease in neural noise impairs scalar property (Buhusi and Oprisan, 2013; Oprisan and Buhusi, 2013a,b, 2014). Because (neural) noise apparently increases rather than decreases as a result of mPFC inactivation, this would further predict that mPFC inactivation should not affect scalar property, as in our study.

Finally, time coding by the coincidental activation of distributed neural inputs also predicts that mPFC inactivation would not affect timing accuracy but would only decrease the precision of timing in a gradual manner. Indeed, this can be understood immediately using a precursor of the SBF model, Miall’s (1989) neural coding model, in which neuronal oscillators code timing similar to a digital/binary code: for example, timing 21 s could be coded by five binary neurons, such that the criterion code for 21 s would be 10101 (21 = 10101 in binary code). In this simple model, if one “inactivates” the second neuron from the right, the “inactivated” system would now respond to the pattern 101x1, which includes 21 s and 23 s (23 = 10111 in binary code) suggesting that: (a) precision would decrease; but (b) the average response (22 s) remains close to the criterion time (21 s). If one now also “inactivates” the 3rd neuron, the system would now respond to the pattern 10xx1, which includes times 17 s, 19 s, 21 s and 23 s, i.e., again: (a) precision would further decrease; but (b) the average response (20 s) would continue to remain relatively close to the original criterion time (21 s), in other words accuracy would be minimally affected. Further inactivation of the 2nd neuron from the right, results in the system responding to the pattern 1xxx1, which includes 17 s, 19 s, 21 s, 23 s, 25 s, 29 s, 31 s, i.e.: (a) precision decreases further; but (b) the average response (24 s) continues to be relatively close to the criterion time (21 s), in other words accuracy would be minimally affected. In summary, such a coding scheme is resistant to input degradation: gradual input degradation results in minimal effect on timing accuracy, but results in a gradual decrease of precision, similar to that observed in our experiment.

**Figure 8:**
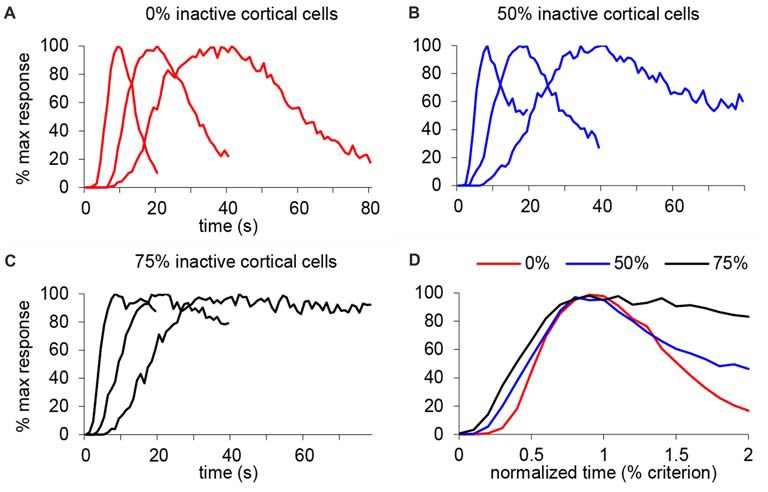
Gradual inactivation of cortical inputs in the Striatal Beat Frequency (SBF) model results in preserved timing accuracy, preserved scalar timing, but gradual impairment in timing precision. Simulated percent maximal SBF output in absolute time units **(A–C)** and normalized time units **(D)** for three criterion durations: 10 s, 20 s and 40 s. **(A)** SBF output when all cortical neurons are active (0% inactive), similar to the saline condition in our study. **(B)** SBF output when only 50% cortical neurons are active (50% inactive) similar to the 7 ug muscimol condition in our study. **(C)** SBF output for only 25% cortical neurons active (75% inactive) similar to the 63 ug muscimol condition in our study. **(D)** Averaged SBF output over the three criteria in normalized time units successfully replicate experimental data presented in Figures 5C,F.

We further tested this idea in an SBF model with oscillatory input neurons (Buhusi and Meck, 2005; Buhusi and Oprisan, 2013), in which the only variable modified was the number of cortical input neurons. Figure 8A shows the response function when all input neurons are active (equivalent to the saline condition in our study), under a mild inactivation of cortical neuronal inputs (50% cortical inactivation, roughly equivalent to the 7 ng/side muscimol condition in our study, Figure 8B), and under a drastic inactivation of neuronal inputs (75% cortical inactivation, roughly equivalent to the 63 ng/side muscimol condition in our study, Figure 8C). As predicted, the SBF model is resistant to input degradation: gradual inactivation of cortical neuronal inputs results in a gradual decrease of timing precision (the response functions become wider, Figure 8D), nevertheless with preservation of timing accuracy and scalar timing.

Considering our results (and the results obtained in paradigms other than timing, discussed below), one final question remains, on whether mPFC activity is specific to timing, or another, more general process, like attention, working memory, or response inhibition/selection. For example, we (Matthews et al., 2012) noted that the presentation of unexpected aversive distractors delays timing in the PI procedure long past the reset boundary, and thus cannot be immediately explained by timing. Yet, mPFC infusion of dopamine-norepinephrine reuptake inhibitor nomifensine reduced the post-distractor delay (Matthews et al., 2012), suggesting that mPFC may be involved in either attention or working memory processes, rather than timing *per se*. Indeed, as a whole, animal and human studies have implicated mPFC in a wide variety of cognitive processes like attention (Arnsten, 2009; Paneri and Gregoriou, 2017), working memory (Funahashi, 2017; Murray et al., 2017; Spaak et al., 2017), inhibitory control (Jonkman et al., 2009), habit formation (Yin and Knowlton, 2006; Limpens et al., 2015), planning and decision making (Dixon and Christoff, 2014; Padoa-Schioppa and Conen, 2017), action selection (Matsumoto et al., 2003; Ridderinkhof et al., 2004) and cognitive flexibility (Robbins, 2007; Kehagia et al., 2010). As in the above studies timing was only an incidental variable (since all events happen in time), mPFC’s involvement in all this body of literature makes highly unlikely that mPFC specifically codes for time, as is sometimes suggested in the literature (Picton et al., 2006; Xu et al., 2014).

In summary, the pattern of interval timing behavior observed following mPFC inactivation includes: (a) preserved timing accuracy; (b) preserved scalar property; (c) a gradual decline in timing precision in proportion to the muscimol dose; and (d) differential changes in response rate during the timed cue and the ITI, with preserved cue-ITI discrimination. To these authors, this pattern seems partly at odds with a coding scheme in which timing is coded solely by ramping mPFC activity, although ramping neuron models have not been clearly extended to cortico-striatal circuits or muscimol, and thus interpretations based on these models are rather speculative. Instead, our results match closely the predictions of a coding scheme in which timing is coded by the coincidental activation of multiple inputs, thus providing support for models using a “distributed” timing code (Miall, 1989; Matell and Meck, 2004; Buhusi and Meck, 2005; Buhusi and Oprisan, 2013). Indeed, recent experimental data indicate that timing-related activity may be also “distributed” in other cortical or sub-cortical areas, including striatum (Bakhurin et al., 2017), amygdala (Dallerac et al., 2017), hippocampus (Eichenbaum, 2014), lateral intra-parietal sulcus (Jazayeri and Shadlen, 2015), and even visual areas (Shuler, 2016). This body of literature provides further evidence for theories proposing that timing is an emergent property of brain-wide coincidence detection at multiple levels (molecular, cellular, local circuit and brain-wide circuits; Buhusi et al., 2016).

## Author Contributions

CB and MB: conceived and designed the experiments and wrote the article. MR: performed the experiment. MR and C-AG: analyzed experimental data. CB and SO: performed computer simulations.

## Conflict of Interest Statement

The authors declare that the research was conducted in the absence of any commercial or financial relationships that could be construed as a potential conflict of interest. The reviewer PER-O and handling Editor declared their shared affiliation.
